# Editorial: Nutrition and oral microbiology: integrative perspectives for improved oral health

**DOI:** 10.3389/froh.2026.1932968

**Published:** 2026-07-29

**Authors:** Veronica Veses, Marinka Mravak-Stipetić, Alba Martínez, Chirag C. Sheth

**Affiliations:** 1 Department of Biomedical Sciences, Universidad CEU Cardenal Herrera, CEU Universities, Alfara del Patriarca, Spain; 2 Faculty of Dental Medicine and Health, Josip Juraj Strossmayer University of Osijek, Osijek, Croatia; 3 Department of Pharmacy, Universidad CEU Cardenal Herrera, CEU Universities, Alfara del Patriarca, Spain; 4 Department of Medicine, Universidad CEU Cardenal Herrera, CEU Universities, Alfara del Patriarca, Spain

**Keywords:** caries, diet, nutrition, oral disease, oral microbiome, probiotics, oral health

## Introduction

The oral microbiome is characterized by eubiosis, a dynamic balance between the host microbial community and immune response, where microorganisms interact to maintain homeostasis of the oral environment. In contrast, dysbiosis represents a disruption of this ecological balance, characterized by changes in the composition and function of the microbial community, linked with increased incidence of oral disease (1).

The oral cavity, being a complex microbial ecosystem, is influenced by dietary patterns, as well as lifestyle factors, which when perturbed, could lead to dysbiosis, impacting the incidence and prevalence of oral pathologies such as caries, periodontal disease, and halitosis (2–4). Recent research identifies diet and supplement use as therapeutic interventions for modulating the oral microbiome and improving oral health outcomes (5, 6).

## Impact of dietary choices on oral health outcomes

Adherence to healthy dietary patterns, including Mediterranean diet, is associated with more diverse and beneficial oral microbiota, while high sugar and carbohydrate intake is linked to increased caries risk (2, 7).

In this special issue, we include a study by Li et al., in which the authors characterize the oral microbiome isolated from three distinct groups of university students (healthy controls, dental caries, and dental fluorosis) with differing preferences for sugar consumption (high and low). The objective of this study was to determine how dietary preference for sweet foods impacted the oral microbiome. The authors found that the overall microbial diversity remained comparable across the study population, however they observed specific significant adaptations to the oral microbiome in a disease and diet specific context.

The article by Wei et al. examined the influence of dietary composition on oral health. The study presents a detailed examination of weekly frequency and quantity of consumption of meat, vegetables, fruit, nuts and dairy items in the diets of a cohort of >4,000 participants. The data were analyzed by multivariate analysis to determine the impact of diet on two key oral health related outcome variables; tooth loss and calculus retention. The authors identified associations between elevated weekly meat intake and tooth loss and vegetable intake frequency with reduced calculus severity. Daily dairy intake, as well as weekly fruit and nut intake were identified as protective against tooth loss, whilst a consumption of 150–500 g of vegetables per day and daily dairy consumption reduced dental calculus severity.

## Modulation of the oral microbiome as a therapeutic strategy

Probiotics can reduce pathogenic bacteria, improve gingival health, decrease halitosis, and partially restore oral microbial homeostasis (3, 6).

In this research topic, we present a systematic review by Inchingolo et al., which analyzes the role of probiotics in caries prevention. Supplementation with *Lactobacillus paracasei*, *Lactobacillus rhamnosus* and *Bifidobacterium lactis* resulted in a significant reduction in the levels of *S. mutans* in the oral cavity. The administration of these probiotics was found to reduce the incidence of new carious lesions and improve the salivary immune profile. The effect of probiotics was transient after discontinuation of supplementation.

The umbrella review of meta-analyses published by Tang et al. summarizes the latest empirical evidence on probiotic use for the modulation and improvement of oral health outcomes. The authors identify *L. rhamnosus* as a key microorganism in caries management and halitosis improvement. The study highlights a high degree of heterogeneity in delivery mechanisms and the strains used, as well as a wide variation in the selection of clinical endpoints. The authors suggest improvements including protocol standardization and delivery enhancement.

We include a study that presents a species-specific investigation into the effect of *Heyndrickxia coagulans* (formerly *Bacillus coagulans*) on two key oral health outcomes, the reduction of *S. mutans* levels and improvement of periodontal indices. Cirio et al. present a systematic review and meta-analysis grouping 7 studies from India, Iran and North Macedonia. The authors reveal broad heterogeneity in treatment conditions and outcome variables measured. The probiotic was delivered as mouthwash, cake and chewable tablets, and implemented in healthy patients as well as high caries risk children. The study findings indicate a potential beneficial use for *H. coagulans* in improving oral health parameters, however further studies are required.

The final article in this research topic is a systematic review and meta-analysis of an innovative therapeutic approach—the application of olive oil for the treatment of periodontal disease. The antioxidant, anti-plaque, anti-inflammatory and antimicrobial properties of olive oil are well documented, and this review analyzes the latest evidence relating to the application of this therapy in improving oral health. The article by López-Valverde et al. highlights the short-term benefits of using olive oil on clinically relevant parameters relating to periodontal health, such as probing depth, gingival attachment and bleeding on probing. The authors demonstrate that olive oil therapy improves patient oral health in the short term, and the results are enhanced further when olive oil is applied as adjunctive therapy as opposed to a single therapy approach.

## Future directions

To conclude, our research topic highlights the fact that oral health interventions that modify the microbiome show potential for improving oral health outcomes across a variety of pathologies and disease conditions. There is increasing evidence to support the empirical use of dietary modifications or supplement use in improving patient outcomes. This demonstrates the need for a greater inter-professional coordination between nutritionists, and primary healthcare providers such as doctors and dentists, to improve understanding of the connection between nutritional choices in order to promote better oral and general health for the population.

Despite promising results, the existing evidence remains limited by significant methodological heterogeneity, including differences in study populations, dietary interventions, probiotic strains, routes of administration, duration of follow-up, and selection of clinical outcomes. Future research should focus on conducting well-designed, adequately powered randomized controlled trials with standardized protocols, clearly defined clinically relevant outcomes, and integrating modern multi-omic approaches to characterize the oral microbiome. Such an approach will allow for a more reliable assessment of the causal relationships between dietary interventions, oral microbiome modulation, and oral and general health outcomes, thereby providing a stronger foundation for the development of personalized preventive and therapeutic strategies. A shift from studies primarily focused on describing the oral microbiome toward rigorously designed interventional research will be essential for translating current knowledge into evidence-based dietary and microbiome-targeted strategies for the prevention and management of oral diseases.

Finally, we would like to emphasize the need for research into the use of dietary modulations on systemic health outcomes via the modulation of the oral microbiome. Some studies are already showing that the oral microbiome can modulate inflammatory cytokines, nutritional status and healthy aging, emphasizing the interconnectedness of oral and systemic health (8, 9).
